# Supplementary material for: Secretagogin expression delineates functionally-specialized populations of striatal parvalbumin-containing interneurons
Source: eLife. 2016 Sep 26;5:e16088. doi: 10.7554/eLife.16088 (PMC5036963; doi:10.7554/eLife.16088)
Supplement: Supplementary file 1. — DOI: http://dx.doi.org/10.7554/eLife.16088.021 [file elife-16088-supp1.docx]

**P-Values in the medio-lateral plane**

| Distance from Bregma (mm) | PV+/Scgn– | PV+/Scgn+ | PV+/Scgn– to PV+/Scgn+ | N numbers (PV+/Scgn–) | N numbers (PV+/Scgn+) |
| --- | --- | --- | --- | --- | --- |
| 2.3 | 0.4005 | 0.7788 | 0.6452 | 171 | 22 |
| 2.0 | 0.1296 | 0.0077 | 0.0646 | 283 | 98 |
| 1.8 | 0.8424 | 0.0006 | 0.0004 | 325 | 86 |
| 1.3 | 0.0736 | 0.0001 | 0.0011 | 356 | 112 |
| 0.8 | 0.4592 | 0.3412 | 0.1566 | 350 | 99 |
| 0.3 | 0.7415 | 0.1944 | 0.1991 | 315 | 82 |
| -0.2 | 0.5208 | 0.0001 | 0.0002 | 256 | 92 |
| -0.7 | 0.0313 | 0.0001 | 0.0001 | 316 | 128 |
| -1.2 | 0.8383 | 0.0001 | 0.0001 | 205 | 115 |
| -1.7 | 0.1962 | 0.0001 | 0.0002 | 165 | 96 |
| -2.0 | 0.9944 | 0.0062 | 0.0542 | 83 | 80 |
| -2.7 | 0.7843 | 0.4530 | 0.5628 | 36 | 109 |
| -3.2 | Not enough values | 0.0639 | Not enough values | Not enough values | 139 |

**P-Values in the Dorso-Ventral plane**

| Distance from Bregma (mm) | PV+/Scgn– | PV+/Scgn+ | PV+/Scgn– to PV+/Scgn+ | N numbers (PV+/Scgn–) | N numbers (PV+/Scgn+) |
| --- | --- | --- | --- | --- | --- |
| 2.3 | 0.3901 | 0.5069 | 0.5690 | 171 | 22 |
| 2.0 | 0.0120 | 0.2250 | 0.8151 | 283 | 98 |
| 1.8 | 0.9307 | 0.4865 | 0.5939 | 325 | 86 |
| 1.3 | 0.0023 | 0.2284 | 0.0151 | 356 | 112 |
| 0.8 | 0.7901 | 0.1567 | 0.1800 | 350 | 99 |
| 0.3 | 0.0217 | 0.3509 | 0.0365 | 315 | 82 |
| -0.2 | 0.0001 | 0.0104 | 0.3774 | 256 | 92 |
| -0.7 | 0.0001 | 0.9709 | 0.0192 | 316 | 128 |
| -1.2 | 0.1127 | 0.0285 | 0.0087 | 205 | 115 |
| -1.7 | 0.0001 | 0.0007 | 0.6925 | 165 | 96 |
| -2.0 | 0.3615 | 0.6054 | 0.6892 | 83 | 80 |
| -2.7 | 0.5210 | 0.3603 | 0.2831 | 36 | 109 |
| -3.2 | Not enough values | 0.0574 | Not enough values | Not enough values | 139 |
