# Supplementary material for: Secretagogin expression delineates functionally-specialized populations of striatal parvalbumin-containing interneurons
Source: eLife. 2016 Sep 26;5:e16088. doi: 10.7554/eLife.16088 (PMC5036963; doi:10.7554/eLife.16088)
Supplement: Supplementary file 2. — DOI: http://dx.doi.org/10.7554/eLife.16088.022 [file elife-16088-supp2.docx]

**P-Values in the Medio-lateral plane of the Caudate**

| Distance from Bregma (mm) | PV+/Scgn– | PV+/Scgn+ | PV+/Scgn– to PV+/Scgn+ | N numbers (PV+/Scgn–) | N numbers (PV+/Scgn+) |
| --- | --- | --- | --- | --- | --- |
| 4.1 | 0.0005 | 0.4418 | 0.0030 | 75 | 171 |
| 2.2 | 0.0110 | 0.3902 | 0.0071 | 160 | 351 |
| 0.0 | 0.8694 | 0.0001 | 0.1960 | 51 | 196 |
| -2.7 | 0.7449 | 0.3881 | 0.8888 | 40 | 130 |
| -5.2 | 0.0077 | 0.0364 | 0.0392 | 34 | 151 |
| -7.6 | 0.0876 | 0.0001 | 0.6200 | 31 | 224 |
| -9.4 | 0.5734 | 0.2036 | 0.8559 | 75 | 341 |

**P-Values in the Medio-lateral plane of the Putamen**

| Distance from Bregma (mm) | PV+/Scgn– | PV+/Scgn+ | PV+/Scgn– to PV+/Scgn+ | N numbers (PV+/Scgn–) | N numbers (PV+/Scgn+) |
| --- | --- | --- | --- | --- | --- |
| 4.1 | 0.0690 | 0.0896 | 0.0059 | 42 | 60 |
| 2.2 | 0.0839 | 0.0298 | 0.4320 | 127 | 317 |
| 0.0 | 0.0001 | 0.4458 | 0.0001 | 145 | 307 |
| -2.7 | 0.0025 | 0.0001 | 0.8667 | 57 | 198 |
| -5.2 | 0.0395 | 0.0331 | 0.3550 | 45 | 180 |
| -7.6 | 0.0020 | 0.2530 | 0.0269 | 147 | 511 |
| -9.4 | 0.0025 | 0.3547 | 0.0278 | 132 | 382 |
